# Supplementary material for: Interobserver agreement for the Chest Wall Injury Society taxonomy of rib fractures using computed tomography images
Source: J Trauma Acute Care Surg. 2022 Aug 31;93(6):736–42. doi: 10.1097/TA.0000000000003766 (PMC9671596; doi:10.1097/TA.0000000000003766)
Supplement: SUPPLEMENTARY MATERIAL [file jt-93-736-s001.docx]

**Background**

In patients who sustained blunt chest trauma, rib fractures are the most common injury (1, 2). Most rib fractures heal with nonoperative therapy (3). Nevertheless, there is a significant burden of morbidity and mortality in many patients with rib fractures, with worse outcomes for those with increasing age or number of rib fractures and presence of flail chest or associated injuries such as pulmonary contusions (4-9).

A vital step for improving care for thoracic trauma patients is providing a universal nomenclature for assessment and communication of chest wall injuries. The utility of such scoring systems for both internal organs and other orthopedic injuries has been described (10). Current scoring systems for rib fractures are the Organ Injury Scale Chest Wall grade, Chest Trauma Score, and chest Abbreviated Injury Scale (11-13). They include the number of fractures, presence of flail chest, and presence of bilateral rib fractures, but do not specify rib fracture characteristics. The RibScore, which is a radiographic rib fracture scoring system based on chest computed tomography (CT), is more specific with additional components that consider fracture displacement and the anatomic area of the rib fracture location (14). The first classification that focuses solely on rib fracture characteristics is based on the Müller AO classification (15, 16) . This classification accounts for rib number, location, fracture type and subtype, and demonstrated a substantial agreement among four reviewers (16).

Most recently the Chest Wall Injury Society (CWIS) published a taxonomy of rib fractures resulting from an international consensus using a Delphi group of 113 respondents (17). It proposes universal definitions for fracture displacement (undisplaced, offset, or displaced) and fracture type classification (simple, wedge, or complex). The location of the fracture on the chest wall is provided in three anatomical sectors (anterior, lateral, or posterior), although no consensus was reached on a universal definition of these anatomic boundaries. The complexity of the rib fracture type and displacement as defined by this taxonomy demonstrated a clinical correlation with pulmonary complications and adverse outcomes (18).

The capability of the taxonomy users to agree on definitions is paramount for the successful use of a universal rib fracture classification. We thus aimed to determine the interobserver agreement on the CWIS taxonomy for rib fractures on images from CT-scans of patients with chest wall injury and to assess if it is influenced by the background of the observers. We hypothesized that there would be at least moderate agreement, regardless of the observers’ background.

# **Methods**

*Study design*

Independent observers who were associated with CWIS, either as a member, or as a colleague of a member who is involved in the care for patients with chest wall injury, were invited by email to evaluate axial, coronal, and sagittal images from 11 computed tomography (CT) scans of rib fractures. An online platform (SurveyMonkey®) was used to execute this survey (19). Multiple reminders to complete the survey were sent by email. The Medical Research Ethics Committee (MEC-2020-0883) exempted the study. No sample size calculations were made. The Guidelines for Reporting Reliability and Agreement Studies (GRRAS) was used to ensure proper reporting of methods, results, and discussion (Supplemental Digital Content 1, <http://links.lww.com/TA/C658>)(20).

*Observers*

A total of 2306 invitations, which included reminder emails, were sent to health care professionals associated with CWIS. The invitation contained a link that provided a single opportunity to fill out the survey to avoid duplicates. The observers were asked to classify type and displacement of rib fractures using the CWIS taxonomy definitions, which were provided in the survey. The CWIS categories for fracture type are: Simple, Wedge and Complex. A simple fracture is defined as one fracture line across the rib, a wedge fracture has a second fracture line which does not span the whole width of the rib, and a complex fracture has at least two fracture lines with one or more fragments spanning the width of the rib. The CWIS categories for fracture displacement are: Undisplaced, Offset and Displaced. Undisplaced fractures are defined where there is at least 90% contact between the cortical surfaces, displaced fractures where there is no cortical contact and offset in between; where there is some cortical contact, but less than 90%. The introduction of the questionnaire provided the definitions and explanatory images with excerpts from the original taxonomy paper for the fracture and dislocation types (17). Observers were asked to indicate the anatomical sectors where the rib fracture was located, based on their own definitions, since no consensus was reached for the definition of the anatomic sectors for the localization of rib fractures in the CWIS taxonomy. At the end of the survey, observers were asked to explain how they distinguished between the anterior, lateral, and posterior rib sectors. Links to illustrations which demonstrated the definitions were provided for reference throughout any portion of the survey.

*Fractures*

The rib fractures included in the survey were identified from an institutional database of adult patients who were admitted and treated for thoracic injury at a level-1 trauma center. The research team selected the final images during a consensus meeting. No clinical information was provided. The selected 11 fractures were from 11 different patients. A set of three images from the chest CT in the axial, sagittal, and coronal plane was uploaded to the internet platform on a single page for every fracture (Figure 1). At least one fracture for each permutation of type, displacement, and location was included.

*Evaluation*

Upon login the observers were asked about their demographics, professional background, number of patients with rib fractures treated by them and their institution, and the details of their clinical practice location. Observers were asked to classify the type of rib fracture, anatomical sector, and dislocation pattern for all cases using the CWIS taxonomy (Figure 1D). The option to leave comments was provided at the end of the survey. The observers could complete the study at their own time and pace.

*Statistical analysis*

All analyses were done using SAS vs. 9.4 (SAS Institute, Cary, NC, USA). All responses were analyzed, including those left incomplete. Categorical variables are presented as frequencies and percentages. Missing values were not imputed. The Fleiss’ multi-rater (Cohen’s) kappa and Gwet’s Agreement Coefficient AC1 were used to calculate agreement among the surgeons concerning rib fracture type, anatomic sector, and displacement. Kappa values and Gwet’s statistics were interpreted as: 0.01–0.20 indicate slight agreement; 0.21–0.40, fair agreement; 0.41–0.60, moderate agreement; 0.61–0.80, substantial agreement; 0.81-0.90, strong agreement; and >0.90 almost perfect agreement (21-24). Both Kappa and AC1 values are provided to ensure the internal validity of the findings, since kappa values can be affected by marginal probability (21).

In order to evaluate potential influencing factors, a stratified analysis was conducted as follows: years of training, surgical specialty, years of work experience, caseload, current continent of practice, number of fellow surgeons at the institution who perform surgical stabilization of rib fractures (SSRF), and if the surgeon was a supervisor of residents or not. In addition, the observer boundaries were added to the stratified analyses used to differentiate between the anatomic sectors for rib fracture location specifically.

Additional analysis was conducted to assess agreement when dichotomizing fracture type into simple versus not simple, which constituted of wedge and complex fractures. Similarly, the fractures were dichotomized into displaced versus not displaced, which included the offset and undisplaced fractures. To further investigate agreement on fracture type and displacement pattern, the cases with less than 80% agreement were identified and qualitatively assessed to determine possible causes of disagreement. Statistical significance was declared where 95% confidence intervals did not overlap.

# **Results**

*Observers*

In total, 90 health care professionals responded to the invitation (4% of total invitations sent, including reminders). Of these 90 participants, 76 (84%) finished the complete survey. Most observers identified as male, North American trauma surgeons with over 10 years of practice experience. The majority reported that they supervised residents (Table 1).

***Interobserver agreement***

*Fracture location*

For fracture location on the anatomic sector of the rib, an overall strong interobserver agreement was found (κ= 0.83 (95% CI 0.69-0.97) AC1 = 0.84 (95% CI 0.81-0.88)), as displayed in Table 2. No statistically significant changes were found when stratified for any of the observer or institutional characteristics (Supplemental Digital Content 2, <http://links.lww.com/TA/C659>).

In order to define the location of the fracture, 24 (32%) observers used the anterior and posterior axillary lines, 17 (22%) used the borders of the serratus anterior, pectoral, and latissimus dorsi muscles, 17 (22%) divided the ribs in equal thirds, six (7.9%) considered the surgical approach that would be used for rib fixation and 12 (16%) did not use a specific method, just experience or “eyeballing”. All methods showed strong agreement for fracture location (κ= 0.81-0.90, AC1= 0.82-0.90), except from the “surgical approach method” which demonstrated only substantial agreement (κ= 0.67 (95% CI 0.46-0.88), AC1=0.71 (95% CI 0.49-0.93). No statistical difference in agreement was found between any of the methods to define the location of the fracture.

*Fracture type*

Interobserver agreement for the CWIS taxonomy definition of fracture type was moderate (κ= 0.46, (95% CI 0.32-0.59), AC1 = 0.50 (95% CI 0.45-0.55)) (Table 2). Observers disagreed most often on the wedge type of fracture (Table 3). When dichotomized in simple versus non-simple fractures, the agreement was somewhat higher, but remained moderate (κ= 0.59 (95% CI 0.42-0.76), AC1 = 0.59 (95% CI 0.53-0.66)) as shown in Supplemental Digital Content 3, <http://links.lww.com/TA/C660>.

After stratifying for observers’ characteristics, no statistically significant differences in agreement on fracture type were found (Supplemental Digital Content 2, <http://links.lww.com/TA/C659>, and 3, <http://links.lww.com/TA/C660>), except from the continent practiced. Agreement was substantial in Europe, moderate in North America, and fair in other continents. Although not statistically significant, agreement was highest, albeit still moderate, in residents, whereas there was only fair agreement for surgeons with more than 20 years’ experience in practice. There was a decreased agreement with lower number of surgeons practicing SSRF in the institution, but not significantly ( Supplemental Digital Content 2, <http://links.lww.com/TA/C659>, and 3, <http://links.lww.com/TA/C660>).

In six out of the 11 cases, over 80% of the observers agreed on the fracture type. In these less complex cases substantial agreement was reached (κ= 0.61 (95% CI 0.43-0.79), AC1 = 0.72 (95% CI 0.65-0.78)). When dichotomizing the type of fractures as simple versus non-simple, only two cases had less than 80% agreement. In the remaining nine cases, interobserver agreement was similarly substantial (κ= 0.68 (95% CI 0.53-0.83), AC1 0.69 (95% CI 0.62-0.76)).

*Fracture displacement*

Interobserver agreement was fair for the CWIS taxonomy definition of undisplaced, offset and displaced fractures (κ=0.38 (95% CI 0.21-0.54), AC1=0.38 (95% CI 0.34-0.42)) (Table 2). When dichotomized in displaced (*i.e.,* offset and displaced) and non-displaced and fractures, agreement was moderate (κ =0.45 (95% CI 0.20-0.71), AC1=0.53 (95% CI0.48-0.58)). Surgeons disagreed most on offset fracture displacement (Table 3).

For agreement in the stratified analysis (Supplemental Digital Content 2, <http://links.lww.com/TA/C659>), the results for fracture displacement were comparable to those of fracture type. No statistically significant differences were found. In the dichotomized analysis statistically significant differences were found for continent practiced, with more agreement in Europe than in North America, and fewest in other continents. Bordering on statistical significance were higher agreement in residents than in surgeons with more than 20 years’ experience in practice, and decreased agreement with lower number of surgeons practicing SSRF in the institution (Supplemental Digital Content 3, <http://links.lww.com/TA/C660>).

In three cases there was over 80% consensus about fracture displacement. In these less complex cases, agreement was substantial to strong (κ =0.76 (95% CI 0.64-0.89), AC1=0.85 (95% CI 0.77-0.92)). When dichotomizing displacement as displaced vs nondisplaced fractures, seven cases reached over 80% consensus, in which agreement was moderate to strong (κ= 0.58 (95% CI 0.19-0.96), AC1=0.81 (95% CI 0.74-0.87)).

***Qualitative analysis***

Qualitative analysis was conducted for the cases with lower than expected (<80%) agreement of fracture type and fracture displacement. Three cases had low agreement on fracture type when using three categories, but higher than 80% agreement when fracture type was dichotomized in simple vs not simple. In one case there was less than 80% agreement on fracture type, regardless of the classification in two or three categories (Table 3).

Four cases had lower than expected agreement for fracture displacement when using three categories, but higher than 80% agreement when fracture displacement was dichotomized in displaced vs nondisplaced. In four other cases agreement was lower than 80% regardless of the classification in two or three categories for displacement (Table 3). These low agreement cases were further evaluated to gain understanding in the reason why agreement was lower than expected, which is described in the Discussion.

# **Discussion**

This study aimed to establish the interobserver agreement on fracture location, type, and displacement as defined by the CWIS taxonomy (17), among a large and diverse group of surgeons in multiple continents who are involved in the care for patients with rib fractures. Strong agreement was found for the classification of the fracture location, and moderate agreement was found for rib fracture type. Fair agreement among observers was found for rib fracture displacement, which is lower than was hypothesized. The interobserver agreement on the classification by Bemelman et al. (16) by four observers was substantial (κ= 0.62 (95% CI 0.59-0.65), which is higher than what was established for the CWIS taxonomy. However, the presented results for the CWIS taxonomy may be more reflective of clinical practice, with over 70 observers from diverse backgrounds. Also, no former experience in the classification of rib fractures was required for participation, nor formal training in the CWIS taxonomy was provided, besides provision of the definitions of the CWIS taxonomy in the introduction and a link to the article.

Agreement on the anatomic location of a rib fracture was strongest of the investigated fracture characteristics, even though multiple methods were used to define anterior, lateral or posterior fractures. There appears to be a clear understanding of location of fracture regardless of the definition used. The possible exception is the surgical approach as the definition, because the least agreement was found among observers who determined location by the surgical approach that would be used for stabilizing the rib fracture. The axillary lines were most often used for determining the location and was associated with high agreement, although it is a surface landmark which can be difficult to establish on single CT images. In the original taxonomy, there was no consensus on the sector boundaries, although the use of axillary lines was also the method that received the most votes. Therefore, based on these combined results, an estimation of the axillary lines might be the recommended method for future studies.

Further qualitative analysis on cases with low agreement on fracture type raised the suspicion that in several cases some of the observes did not see all the fracture lines in complex fractures. This probably resulted in a spread between wedge and complex type fractures. Additionally, in two cases with low agreement on type, the fracture did not completely fit with the CWIS taxonomy definitions. One fracture consisted of one rib-width fracture line and multiple incomplete fracture lines, creating two butterfly fragments (Figure 2). Most observers (54%) responded that this was a wedge type fracture, suggesting that a fracture with multiple butterfly fragments should probably classified as such. In another case, there were two rib width fracture lines with a considerable space in between. No specific CWIS taxonomy definition exists for the maximum distance between multiple fracture lines to be considered either one wedge or complex fracture or two separate fractures. This resulted in low agreement on type for this case. We suggest estimating if the fragment could be part of a flail pattern or not, which practically would be around a maximum of 2 centimeters between the fracture lines to be still considered the same fracture, based on our expert opinion.

Furthermore, the qualitative analysis demonstrated that agreement on rib fracture displacement was lowest in complex type fractures. With multiple fracture lines, it seemed to vary if observers would grade the fracture line with the least or the most cortical bone contact, or if they made an overall estimate of bone contact. Moreover, in some cases it was suspected that a portion of the observers accounted for alignment rather than cortical bone contact of the fracture. In one case a butterfly segment was completely displaced, with the contralateral cortex still in place. In this scenario, 49% of observers considered this a displaced fracture, whereas 51% evaluated this as offset. This suggests that agreement on displacement is typically lower for fractures that consist of more than one fracture line. It seems insufficiently clear in the CWIS taxonomy definition how to establish displacement in fractures with multiple fragments. Probably considering the cortical bone contact of both ends of the fracture rather than alignment should be the preferred method, because this probably reflects the instability of the fracture better, as displacement can worsen over time (24). Additionally, the stratified analysis demonstrated that health care providers from Europe agreed more than health care providers from other continents, without a possible explanation that could be supported by the collected data. Theoretically, the difference might reflect a more homogenous group of European participants compared to health care providers from other parts of the world, although again, this theory is not supported by the collected data.

The CWIS taxonomy consist of three categories for rib fracture type and displacement. In theory, agreement improves by reducing the number of categories, based on chance alone. However, when the categories for fracture type and displacement were dichotomized, in “simple vs not simple” and “displaced vs not displaced”, the agreement improved somewhat, although this was only statistically significant in the AC1 for fracture displacement. Moreover, the clinical significance of categorizing in three rib fracture types is not clear, since only complex fractures are associated with worse outcomes (18). Potentially it is sufficient to categorize rib fracture type in only in “simple” and “complex”, not discerning wedge type fractures as a separate category from the simple type since agreement and clinical relevance in this category is low. However, for fracture displacement, three categories are probably justified. This is because the three fracture displacement categories are associated with clinical outcomes and displacement is commonly used to set the indication for surgery (18). However, the offset category had the least agreement in the displacement category, warranting a further specification of the definition. For example, it is currently unclear if offset is defined as between 10% and 90% bone contact in one image or if it should be visible in multiple images, and in just one plane, e.g. transversal, or at least one other plane. This should be better defined and evaluated in future studies accounting for rib fracture characteristics.

Over the past few years, CT reconstructions in 3D and unfolded reconstructions of the chest wall are becoming more widely available (25, 26). It is yet to be determined what the interobserver agreement will be for the classification of rib fractures aided with these imaging modalities. To evaluate their contribution, future interobserver studies will be necessary.

This study has some limitations. Most importantly, the provided imaging was limited in comparison to daily practice for practical and confidentiality reasons. Providing the whole CT-scan with the possibility to change the settings, use zoom options and enhance contrast, might have resulted in more heterogeneity in the responses, lowering agreement. Second, a learning curve for applying the taxonomy definitions could have been present but was not accounted for, although not observed. Moreover, it is unknown how often observers had previously participated in this type of study or in the Delphi group developing the CWIS taxonomy. Third, it is unclear if absence of clinical information has influenced the results. Possibly a history of high energetic thoracic trauma could stimulate the observer to select a more severe injury type and displacement.

Despite these limitations, this study suggests that the CWIS taxonomy has strong agreement on fracture location, moderate agreement on fracture type, and fair agreement on fracture displacement. Revisiting the definitions of the CWIS taxonomy on rib fracture type and displacement may be warranted. For rib fracture type, the “wedge” category could potentially be omitted. For rib fracture displacement, the percentage of bone contact rather than alignment should be clearer in the definition, especially for rib fractures with multiple fragments. Nevertheless, these changes will have to be evaluated. The role of additional or enhanced imaging from the chest CT-scans by 3D reconstructions will have to be assessed for its ability to increase agreement on the classification of fracture type and displacement as defined by the CWIS taxonomy.

# **Supplemental Digital Content**

**SDC 1.** Guidelines for Reporting Reliability and Agreement Studies (GRRAS) checklist

**SDC 2.** Stratified analysis of interobserver agreement on fracture location, type, and displacement

**SDC 3.**  Stratified analysis for interobserver agreement on fracture type and fracture displacement with two categories combined

# **References**

1. Ziegler DW, Agarwal NN. The morbidity and mortality of rib fractures. *J Trauma*. 1994;37(6):975-9.

2. Sirmali M, Türüt H, Topçu S, Gülhan E, Yazici U, Kaya S, et al. A comprehensive analysis of traumatic rib fractures: morbidity, mortality and management. *Eur J Cardiothorac Surg*. 2003;24(1):133-8.

3. Pieracci FM, Majercik S, Ali-Osman F, Ang D, Doben A, Edwards JG, et al. Consensus statement: Surgical stabilization of rib fractures rib fracture colloquium clinical practice guidelines. *Injury*. 2017;48(2):307-21.

4. Flagel BT, Luchette FA, Reed RL, Esposito TJ, Davis KA, Santaniello JM, et al. Half-a-dozen ribs: the breakpoint for mortality. *Surgery*. 2005;138(4):717-23; discussion 23-5.

5. Holcomb JB, McMullin NR, Kozar RA, Lygas MH, Moore FA. Morbidity from rib fractures increases after age 45. *J Am Coll Surg*. 2003;196(4):549-55.

6. Rostas JW, Lively TB, Brevard SB, Simmons JD, Frotan MA, Gonzalez RP. Rib fractures and their association With solid organ injury: higher rib fractures have greater significance for solid organ injury screening. *Am J Surg*. 2017;213(4):791-7.

7. Liman ST, Kuzucu A, Tastepe AI, Ulasan GN, Topcu S. Chest injury due to blunt trauma. *Eur J Cardiothorac Surg*. 2003;23(3):374-8.

8. Bergeron E, Lavoie A, Clas D, Moore L, Ratte S, Tetreault S, et al. Elderly trauma patients with rib fractures are at greater risk of death and pneumonia. *J Trauma*. 2003;54(3):478-85.

9. Pape HC, Remmers D, Rice J, Ebisch M, Krettek C, Tscherne H. Appraisal of early evaluation of blunt chest trauma: development of a standardized scoring system for initial clinical decision making. *J Trauma*. 2000;49(3):496-504.

10. Lecky F, Woodford M, Edwards A, Bouamra O, Coats T. Trauma scoring systems and databases. *Br J Anaesth*. 2014;113(2):286-94.

11. Pressley CM, Fry WR, Philp AS, Berry SD, Smith RS. Predicting outcome of patients with chest wall injury. *Am J Surg*. 2012;204(6):910-3; discussion 3-4.

12. Moore EE, Cogbill TH, Jurkovich GJ, McAninch JW, Champion HR, Gennarelli TA, et al. Organ injury scaling. III: Chest wall, abdominal vascular, ureter, bladder, and urethra. *J Trauma*. 1992;33(3):337-9.

13. Baker JE, Millar DA, Heh V, Goodman MD, Pritts TA, Janowak CF. Does chest wall Organ Injury Scale (OIS) or Abbreviated Injury Scale (AIS) predict outcomes? An analysis of 16,000 consecutive rib fractures. *Surgery*. 2020;168(1):198-204.

14. Chapman BC, Herbert B, Rodil M, Salotto J, Stovall RT, Biffl W, et al. RibScore: A novel radiographic score based on fracture pattern that predicts pneumonia, respiratory failure, and tracheostomy. *J Trauma Acute Care Surg*. 2016;80(1):95-101.

15. Müller ME, Heim U, Nazarian S, Koch P, Schatzker J. The Comprehensive Classification of Fractures of Long Bones: Springer Berlin Heidelberg; 1990.

16. Bemelman M, Baal MV, Raaijmakers C, Lansink K, Leenen L, Long W. An Interobserver Agreement Study with a New Classification for Rib Fractures. *Chirurgia* (Bucur). 2019;114(3):352-8.

17. Edwards JG, Clarke P, Pieracci FM, Bemelman M, Black EA, Doben A, et al. Taxonomy of multiple rib fractures: Results of the chest wall injury society international consensus survey. *J Trauma Acute Care Surg*. 2020;88(2):e40-e5.

18. Clarke PTM, Simpson RB, Dorman JR, Hunt WJ, Edwards JG. Determining the clinical significance of the Chest Wall Injury Society taxonomy for multiple rib fractures. *J Trauma Acute Care Surg*. 2019;87(6):1282-8.

19. SurveyMonkey Inc.: San Mateo, California, USA; 2021 [Available from: [www.surveymonkey.com](file:///\\storage.erasmusmc.nl\v\vcl05\HEEL\DATA\USERS\FixCon\FixCon%20Suzanne%20van%20Wijck\05%20TICC-Tax\JTACS%20submission\R1%20JTACS\www.surveymonkey.com).]

20. Kottner J, Audigé L, Brorson S, Donner A, Gajewski BJ, Hróbjartsson A, et al. Guidelines for Reporting Reliability and Agreement Studies (GRRAS) were proposed. *J Clin Epidemiol*. 2011;64(1):96-106.

21. Wongpakaran N, Wongpakaran T, Wedding D, Gwet KL. A comparison of Cohen's Kappa and Gwet's AC1 when calculating inter-rater reliability coefficients: a study conducted with personality disorder samples. *BMC Med Res Methodol*. 2013;13:61.

22. Cohen J. A Coefficient of Agreement for Nominal Scales. *Educ Psychol Meas*. 1960;20(1):37-46.

23. Fleiss JL, Cohen J. The Equivalence of Weighted Kappa and the Intraclass Correlation Coefficient as Measures of Reliability. *Educ Psychol Meas*. 1973;33(3):613-9.

24. McHugh ML. Interrater reliability: the kappa statistic. *Biochem Med* (Zagreb). 2012;22(3):276-82.

25. Khung S, Masset P, Duhamel A, Faivre JB, Flohr T, Remy J, et al. Automated 3D Rendering of Ribs in 110 Polytrauma Patients: Strengths and Limitations. *Acad Radiol.* 2017;24(2):146-52.

26. Pulley BR, Taylor BC, Fowler TT, Dominguez N, Trinh TQ. Utility of three-dimensional computed tomography for the surgical management of rib fractures. *J Trauma Acute Care Surg*. 2015;78(3):530-4.

**Figure legends**

# **Figure 1.** Example of a rib fracture case included in the survey, with A) the axial plane, B) the sagittal plane, C) the coronal plane, and D) the survey questions

The fracture is indicated with the yellow circle

# **Figure 2.** Transverse view of a low agreement case on type of fracture; there is one rib-width fracture line and there are two fracture lines which do not span the whole width

The fracture lines are indicated in red

# **Table 1.** Observer characteristics

| **Variables** | **N*** | **Number of observers (%)** |
| --- | --- | --- |
| Gender Male | 88 | 75 (85%) |
| Female |  | 11 (13%) |
| Prefer not to answer |  | 2 (2%) |
| Continent North America | 90 | 55 (61%) |
| Europe |  | 20 (22%) |
| South America |  | 6 (7%) |
| Australia/New Zealand |  | 4 (4%) |
| Asia |  | 4 (4%) |
| Africa |  | 1 (1%) |
| Surgical specialty Trauma | 90 | 61 (68%) |
| Thoracic |  | 17 (19%) |
| Orthopedic |  | 6 (7%) |
| General |  | 3 (3%) |
| Cardiothoracic |  | 1 (1%) |
| Pediatric |  | 1 (1%) |
| Combination of trauma and thoracic |  | 1 (1%) |
| Experience in practice Resident | 90 | 8 (9%) |
| Less than 5 years |  | 19 (21%) |
| 6-10 years |  | 23 (26%) |
| 11-20 years |  | 31 (34%) |
| Greater than 20 years |  | 9 (10%) |
| Resident supervision Yes | 90 | 77 (86%) |
| No |  | 13 (14%) |
| Patients with rib fractures treated annually ≤ 5 patients | 90 | 7 (8%) |
| 6-10 patients |  | 4 (4%) |
| 11-20 patients |  | 11 (12%) |
| ≥ 21 patients |  | 68 (76%) |
| Institutional annual volume of rib fracture patients ≤ 50 | 90 | 11 (12%) |
| 51-100 |  | 15 (17%) |
| 101-150 |  | 17 (19%) |
| 151-200 |  | 12 (13%) |
| ≥ 201 |  | 35 (39%) |
| Number of surgeons performing SSRF at observer’s institution | 90 |  |
| 1 |  | 13 (14%) |
| 2 |  | 18 (20%) |
| 3 |  | 21 (23%) |
| 4 |  | 15 (17%) |
| ≥5 |  | 23 (26%) |

Data are shown as number of observers (% of total)

* N is the number of complete responses

SSRF, surgical stabilization of rib fractures

# **Table 2.** Interobserver agreement for the classification of rib fractures based on the CWIS taxonomy

|  | **N*** | **Κ (95% CI)** | **AC1 (95% CI)** | **Interpretation of agreement** |
| --- | --- | --- | --- | --- |
| Fracture location | 75 | 0.83 (0.69-0.97) | 0.84 (0.81-0.88) | Strong |
| Fracture type | 76 | 0.46 (0.32-0.59) | 0.50 (0.45-0.55) | Moderate |
| Fracture displacement | 76 | 0.38 (0.21-0.54) | 0.38 (0.34-0.42) | Fair |

Data are shown as unweighted Κ and AC1 scores with (95% confidence interval)

* N is the number of complete responses

AC1, Gwet’s first agreement coefficient; CI, confidence interval; K, kappa value

**Table 3.** Rib fracture case agreement on type and displacement

|  | **N*** | **Fracture type** |  |  | **Fracture displacement** | |  |
| --- | --- | --- | --- | --- | --- | --- | --- |
| **Case** |  | **Simple** | **Wedge** | **Complex** | **Displaced** | **Offset** | **Undisplaced** |
| #1 ^D^ | 84 | 67 (80%) | 8 (10%) | 9 (11%) | 63 (75%) | 15 (18%) | 6 (7%) |
| #2 ^A+C^ | 80 | 9 (11%) | 11 (14%) | 60 (75%) | 11 (14%) | 44 (54%) | 26 (32%) |
| #3 ^A+C^ | 80 | 9 (11%) | 43 (54%) | 28 (35%) | 8 (10%) | 26 (33%) | 46 (58%) |
| #4^D^ | 80 | 71 (89%) | 4 (5%) | 5 (6%) | 29 (36%) | 15 (19%) | 36 (45%) |
| #5 | 77 | 59 (77%) | 5 (7%) | 13 (17%) | 71 (92%) | 4 (5%) | 2 (3%) |
| #6 ^D^ | 77 | 1 (1%) | 6 (8%) | 70 (91%) | 55 (71%) | 18 (23%) | 4 (5%) |
| #7 ^B+D^ | 77 | 35 (46%) | 26 (34%) | 16 (21%) | 38 (49%) | 39 (51%) | 0 (0%) |
| #8 ^A+C^ | 76 | 12(16%) | 48 (63%) | 16 (21%) | 5 (7%) | 47 (62%) | 24 (32%) |
| #9 | 76 | 73 (96%) | 3 (4%) | 0 (0%) | 0 (0%) | 6 (8%) | 70 (92%) |
| #10 | 76 | 73 (96%) | 2 (3%) | 1 (1%) | 1 (1%) | 1 (1%) | 74 (97%) |
| #11 ^C^ | 76 | 0 (0%) | 15 (20%) | 61 (80%) | 9 (12%) | 45 (59%) | 22 (29%) |
| Total | 859 | 409 | 171 | 279 | 290 | 260 | 310 |

Data are shown as number of observers (% agreement)

* N is the number of complete responses

^A^ Cases with <80% agreement in three categories, with ≥80% agreement when combined in two categories for fracture type

^B^ Cases with <80% agreement in three categories and when combined in two categories for fracture type

^C^ Cases with <80% agreement in three categories, with ≥80% agreement when combined in two categories for fracture displacement

^D^ Cases with <80% agreement in three categories and when combined in two categories for fracture displacement
