# Supplementary material for: Interobserver agreement for the Chest Wall Injury Society taxonomy of rib fractures using computed tomography images
Source: J Trauma Acute Care Surg. 2022 Aug 31;93(6):736–42. doi: 10.1097/TA.0000000000003766 (PMC9671596; doi:10.1097/TA.0000000000003766)
Supplement: SUPPLEMENTARY MATERIAL [file jt-93-736-s002.docx]

| Section/topic |  | Item | Manuscript Page |
| --- | --- | --- | --- |
| TITLE AND  ABSTRACT | 1. | Identify in title or abstract that interrater/intrarater reliability or agreement was investigated. | 1 and abstract |
| INTRODUCTION | 2. | Name and describe the diagnostic or measurement device of interest explicitly. | 1 and 2 |
|  | 3. | Specify the subject population of interest. | 1 |
|  | 4. | Specify the rater population of interest (if applicable). | 2 |
|  | 5. | Describe what is already known about reliability and agreement and provide a rationale for the study (if applicable). | 2 |
| METHODS | 6. | Explain how the sample size was chosen. State the determined number of raters, subjects/objects, and replicate observations. | 3 |
|  | 7. | Describe the sampling method. | 3 and 4 |
|  | 8. | Describe the measurement/rating process (e.g. time interval between repeated measurements, availability of clinical information, blinding). | 3 and 4 |
|  | 9. | State whether measurements/ratings were conducted independently. | 4 |
|  | 10. | Describe the statistical analysis. | 5 and 6 |
| RESULTS | 11. | State the actual number of raters and subjects/objects which were included and the number of replicate observations which were conducted. | 7 |
|  | 12. | Describe the sample characteristics of raters and subjects (e.g. training, experience). | 7 and table 1 |
|  | 13. | Report estimates of reliability and agreement including measures of statistical uncertainty. | 7,8,9 and table 2,3, SDC 2 and SDC 3 |
| DISCUSSION | 14. | Discuss the practical relevance of results. | 11 and 12 |
| AUXILIARY  MATERIAL | 15. | Provide detailed results if possible (e.g. online) | Not applicable |

**Supplemental Digital Content 1.** Guidelines for Reporting Reliability and Agreement Studies (GRRAS) checklist
