# Supplementary material for: Interobserver agreement for the Chest Wall Injury Society taxonomy of rib fractures using computed tomography images
Source: J Trauma Acute Care Surg. 2022 Aug 31;93(6):736–42. doi: 10.1097/TA.0000000000003766 (PMC9671596; doi:10.1097/TA.0000000000003766)
Supplement: SUPPLEMENTARY MATERIAL [file jt-93-736-s003.docx]

**Supplemental Digital Content 2.** Stratified analysis of interobserver agreement on fracture location, type, and displacement

|  | **Anatomic location** | | | **Type** | | | | | **Displacement** | | | |
| --- | --- | --- | --- | --- | --- | --- | --- | --- | --- | --- | --- | --- |
|  | **Κ (95% CI)** | **AC1 (95% CI)** | | **Κ (95% CI)** | | **AC1 (95% CI)** | | | **Κ (95% CI)** | | | **AC1 (95% CI)** |
| Overall agreement | 0.83 (0.69-0.97) | 0.84 (0.81-0.88) | | 0.46 (0.32-0.59) | | 0.50 (0.45-0.55) | | | 0.38 (0.21-0.54) | | | 0.38 (0.34-0.42) |
| Specialty |  |  | |  | |  | | |  | | |  |
| Trauma | 0.84 (0.71-0.97) | 0.86 (0.81-0.90) | | 0.49 (0.35-0.63) | | 0.54 (0.48-0.60) | | | 0.41 (0.24-0.57) | | | 0.41 (0.36-0.46) |
| Not trauma | 0.81 (0.64-0.98) | 0.83 (0.76-0.89) | | 0.39 (0.25-0.53) | | 0.43 (0.34-0.52) | | | 0.30 (0.14-0.47) | | | 0.31 (0.24-0.39) |
| Continent |  |  | |  | |  | | |  | | |  |
| Europe | 0.83 (0.68-0.98) | 0.84 (0.77-0.91) | | 0.64 (0.49-0.79) | | 0.67 (0.56-0.78) | | | 0.45 (0.25-0.64) | | | 0.45 (0.36-0.54) |
| North America | 0.82 (0.69-0.96) | 0.84 (0.79-0.89) | | 0.46 (0.32-0.60) | | 0.51 (0.45-0.57) | | | 0.38 (0.22-0.54) | | | 0.38 (0.33-0.43) |
| Other | 0.84 (0.68-1.00) | 0.85 (0.78-0.92) | | 0.29 (0.12-0.46) | | 0.32 (0.22-0.43) | | | 0.29 (0.10-0.48) | | | 0.31 (0.22-0.41) |
| Years in practice |  |  | |  | |  | | |  | | |  |
| Resident | 0.79 (0.56-1.01) | 0.80 (0.63-0.97) | | 0.52 (0.33-0.72) | | 0.55 (0.28-0.82) | | | 0.47 (0.27-0.67) | | | 0.51 (-0.01-1.00) |
| <5 years | 0.84 (0.68-1.00) | 0.86 (0.78-0.93) | | 0.46 (0.31-0.61) | | 0.52 (0.39-0.65) | | | 0.34 (0.19-0.49) | | | 0.34 (0.28-0.41) |
| 6-10 years | 0.77 (0.62-0.91) | 0.79 (0.69-0.89) | | 0.50 (0.35-0.65) | | 0.55 (0.44-0.66) | | | 0.34 (0.18-0.51) | | | 0.35 (0.23-0.46) |
| 11-20 years | 0.89 (0.74-1.03) | 0.90 (0.86-0.93) | | 0.44 (0.30-0.59) | | 0.49 (0.40-0.57) | | | 0.41 (0.22-0.60) | | | 0.42 (0.35-0.49) |
| >20 years | 0.77 (0.57-0.97) | 0.79 (0.66-0.92) | | 0.33 (0.10-0.56) | | 0.37 (0.25-0.50) | | | 0.31 (0.08-0.53) | | | 0.31 (0.14-0.48) |
| Supervisor of residents |  |  | |  | |  | | |  | | |  |
| Yes | 0.83 (0.69-0.97) | 0.85 (0.81-0.89) | | 0.47 (0.33-0.60) | | 0.51 (0.46-0.57) | | | 0.38 (0.21-0.54) | | | 0.38 (0.34-0.42) |
| No | 0.80 (0.61-0.98) | 0.81 (0.68-0.94) | | 0.37 (0.18-0.55) | | 0.41 (0.26-0.55) | | | 0.33 (0.13-0.54) | | | 0.34 (0.15-0.53) |
| Total of SSRF performed | |  | |  | |  | | |  | | |  |
| >50 cases | 0.84 (0.69-0.98) | 0.85 (0.79-0.90) | | 0.43 (0.28-0.58) | | 0.47 (0.40-0.55) | | | 0.37 (0.19-0.55) | | | 0.38 (0.33-0.44) |
| ≤50 cases | 0.82 (0.68-0.97) | 0.84 (0.79-0.89) | | 0.48 (0.34-0.62) | | 0.53 (0.46-0.60) | | | 0.38 (0.23-0.53) | | | 0.38 (0.31-0.45) |
| Observer caseload |  |  | |  | |  | | |  | | |  |
| >20 patients/y | 0.82 (0.68-0.96) | 0.84 (0.79-0.88) | | 0.46 (0.31-0.60) | | 0.50 (0.44-0.56) | | | 0.38 (0.22-0.54) | | | 0.38 (0.33-0.43) |
| ≤20 patients/y | 0.85 (0.69-1.01) | 0.86 (0.81-0.91) | | 0.45 (0.29-0.61) | | 0.50 (0.37-0.63) | | | 0.40 (0.19-0.60) | | | 0.41 (0.31-0.51) |
| Institutional volume of rib fracture patients | |  | | |  | | |  | | |  | |
| >200 patients/y | 0.82 (0.69-0.94) | 0.83 (0.76-0.90) | | 0.48 (0.32-0.63) | | 0.52 (0.44-0.60) | | | 0.38 (0.22-0.54) | | | 0.38 (0.32-0.44) |
| ≤200 patients/y | 0.84 (0.69-1.00) | 0.86 (0.82-0.90) | | 0.43 (0.31-0.56) | | 0.48 (0.42-0.55) | | | 0.37 (0.20-0.55) | | | 0.38 (0.33-0.44) |
| No. of surgeons performing SSRF in institution | | |  | | | |  | | |  | | |
| ≥5 surgeons | 0.81 (0.67-0.95) | 0.83 (0.73-0.93) | | 0.50 (0.33-0.66) | | 0.55 (0.42-0.68) | | | 0.39 (0.20-0.58) | | | 0.40 (0.33-0.47) |
| 3-4 surgeons | 0.84 (0.67-1.00) | 0.85 (0.81-0.89) | | 0.50 (0.37-0.64) | | 0.54 (0.46-0.62) | | | 0.39 (0.22-0.56) | | | 0.39 (0.32-0.47) |
| 1-2 surgeons | 0.84 (0.69-0.99) | 0.85 (0.79-0.92) | | 0.36 (0.20-0.52) | | 0.40 (0.32-0.48) | | | 0.34 (0.20-0.48) | | | 0.35 (0.27-0.42) |

Data are shown as unweighted Κ and AC1 scores with (95% confidence interval)

AC1, Gwet’s first agreement coefficient; CI, confidence interval; K, kappa value; SSRF, surgical stabilization of rib fractures
