# Supplementary material for: Interobserver agreement for the Chest Wall Injury Society taxonomy of rib fractures using computed tomography images
Source: J Trauma Acute Care Surg. 2022 Aug 31;93(6):736–42. doi: 10.1097/TA.0000000000003766 (PMC9671596; doi:10.1097/TA.0000000000003766)
Supplement: SUPPLEMENTARY MATERIAL [file jt-93-736-s004.docx]

**Supplemental Digital Content 3.** Stratified analysis for interobserver agreement on fracture type and fracture displacement with two categories combined

|  | **Type** | | | | **Displacement** | |
| --- | --- | --- | --- | --- | --- | --- |
|  | **Κ (95% CI)** | | **AC1 (95% CI)** | | **Κ (95% CI)** | **AC1 (95% CI)** |
| Overall agreement with 2 scoring categories (simple/not simple, displaced/not displaced) | 0.59 (0.42-0.76) | | 0.59 (0.53-0.66) | | 0.45 (0.20-0.71) | 0.53 (0.48-0.58) |
| Specialty |  | |  | |  |  |
| Trauma | 0.61 (0.44-0.77) | | 0.61 (0.54-0.68) | | 0.46 (0.20-0.72) | 0.54 (0.48-0.60) |
| Not trauma | 0.56 (0.36-0.76) | | 0.56 (0.43-0.69) | | 0.42 (0.16-0.69) | 0.50 (0.41-0.59) |
| Continent |  | |  | |  |  |
| Europe | 0.72 (0.54-0.91) | | 0.73 (0.64-0.81) | | 0.55 (0.26-0.84) | 0.61 (0.48-0.74) |
| North America | 0.60 (0.43-0.77) | | 0.60 (0.52-0.68) | | 0.44 (0.19-0.69) | 0.51 (0.45-0.57) |
| Other | 0.43 (0.19-0.67) | | 0.45 (0.29-0.60) | | 0.39 (0.09-0.70) | 0.52 (0.38-0.67) |
| Years in practice |  | |  | |  |  |
| Resident | 0.73 (0.47-1.00) | | 0.74 (0.53-0.95) | | 0.54 (0.27-0.81) | 0.58 (0.22-0.93) |
| <5 years | 0.59 (0.39-0.78) | | 0.59 (0.44-0.73) | | 0.37 (0.13-0.60) | 0.44 (0.36-0.52) |
| 6-10 years | 0.64 (0.47-0.81) | | 0.64 (0.50-0.78) | | 0.46 (0.22-0.71) | 0.55 (0.43-0.66) |
| 11-20 years | 0.57 (0.39-0.75) | | 0.58 (0.47-0.68) | | 0.49 (0.19-0.79) | 0.56 (0.46-0.65) |
| >20 years | 0.49 (0.21-0.78) | | 0.51 (0.25-0.76) | | 0.39 (0.04-0.73) | 0.56 (0.41-0.71) |
| Supervisor of residents |  | |  | |  |  |
| Yes | 0.60 (0.43-0.77) | | 0.60 (0.53-0.67) | | 0.46 (0.19-0.73) | 0.53 (0.48-0.59) |
| No | 0.54 (0.30-0.78) | | 0.55 (0.38-0.72) | | 0.41 (0.16-0.66) | 0.49 (0.30-0.68) |
| Total of SSRF performed |  | |  | |  |  |
| >50 cases | 0.58 (0.40-0.75) | | 0.58 (0.48-0.68) | | 0.46 (0.17-0.75) | 0.53 (0.46-0.59) |
| ≤50 cases | 0.60 (0.42-0.78) | | 0.60 (0.51-0.69) | | 0.45 (0.22-0.68) | 0.54 (0.46-0.62) |
| Observer caseload |  | |  | |  |  |
| >20 patients/year | 0.59 (0.42-0.75) | | 0.59 (0.52-0.66) | | 0.46 (0.21-0.71) | 0.54 (0.48-0.60) |
| ≤20 patients/year | 0.60 (0.38-0.82) | | 0.60 (0.42-0.78) | | 0.45 (0.15-0.74) | 0.50 (0.40-0.60) |
| Institutional volume of rib fracture patients | | | |  | | |
| >200 patients/year | 0.63 (0.45-0.80) | | 0.63 (0.53-0.73) | | 0.46 (0.19-0.73) | 0.58 (0.52-0.64) |
| ≤200 patients/year | 0.56 (0.39-0.73) | | 0.56 (0.47-0.65) | | 0.46 (0.20-0.71) | 0.50 (0.44-0.57) |
| No. of surgeons performing SSRF in institution | |  |  |  |  |  |
| ≥5 surgeons | 0.65 (0.45-0.84) | | 0.65 (0.49-0.80) | | 0.48 (0.19-0.77) | 0.51 (0.42-0.61) |
| 3-4 surgeons | 0.62 (0.46-0.79) | | 0.63 (0.52-0.73) | | 0.47 (0.19-0.75) | 0.57 (0.50-0.64) |
| 1-2 surgeons | 0.50 (0.28-0.72) | | 0.51 (0.42-0.60) | | 0.42 (0.19-0.65) | 0.50 (0.40-0.61) |

Data are shown as unweighted Κ and AC1 scores with (95% confidence interval)

AC1, Gwet’s first agreement coefficient; CI, confidence interval; K, kappa value; SSRF, surgical stabilization of rib fractures
